# Psychological Safety as a Predictor of Acute Stress, Well-Being and Burnout in Health and Social Care Workers: A Predictive Correlational Study

**DOI:** 10.3390/bs16030418

**Published:** 2026-03-12

**Authors:** Nicola Cogan, Martin Smith, Karen Deakin

**Affiliations:** 1 Department of Psychological Sciences and Health, University of Strathclyde, Graham Hills Building, Glasgow G1 1QE, UK; 2 NHS Lanarkshire, Psychological Services, 50 Netherton Street, Wishaw ML2 0DP, UK

**Keywords:** psychological safety, acute stress, burnout, well-being, health and social care workers, mental health, Polyvagal Theory

## Abstract

**Background:** Health and social care workers (HSCWs) experience high levels of stress, burnout and emotional strain. Psychological safety is increasingly recognised as a protective factor, yet limited research has examined how individual psychological safety and team psychological safety jointly contribute to mental health outcomes in frontline care environments. **Methods:** A total of 821 HSCWs completed validated measures assessing individual psychological safety (NPSS), team psychological safety (TPSS), well-being, burnout and acute stress. Correlational analyses and hierarchical regression models were used to evaluate the unique and shared contributions of individual and team psychological safety to each outcome. **Results:** Both NPSS and TPSS were significantly associated with well-being, burnout and acute stress. Team psychological safety emerged as the strongest predictor of burnout and acute stress, accounting for substantial additional variance beyond individual psychological safety, with increases in explained variance ranging from 0.14 to 0.16. For well-being, NPSS (β = 0.38) and TPSS (β = 0.36) were both significant predictors. TPSS demonstrated large effects for burnout (β = 0.67) and acute stress (β = 0.72). **Conclusions:** Psychological safety plays a central role in the mental health of HSCWs. Team-based psychological safety was particularly influential in protecting against burnout and acute stress, while individual neuroceptive safety remained important for supporting overall well-being. Strengthening both individual and team-level psychological safety may enhance resilience and reduce psychological risk within health and social care settings. **Implications:** Incorporating NPSS and TPSS within workforce assessment may support early identification of psychological vulnerability, inform trauma-informed organisational interventions and promote more resilient, supportive workplace cultures.

## 1. Introduction

Health and social care workers (HSCWs) operate in environments characterised by sustained pressure, high emotional labour, and frequent exposure to traumatic or morally distressing events. These conditions contribute to elevated levels of burnout, acute stress, and reduced psychological well-being across global health and social care systems (Cogan et al., 2022b; Cogan et al., 2022a; Fronteira et al., 2024; Jun et al., 2021). Workforce challenges including chronic understaffing, high sickness absence, and increased turnover have intensified in recent years, placing additional strain on services and heightening the need for effective strategies to support the resilience and mental health of those delivering care. Psychological safety has emerged as a critical protective factor within high-risk, high-demand settings (Peddie et al., 2025). Traditionally conceptualised at the team level, psychological safety refers to a shared belief that it is safe to take interpersonal risks such as asking questions, raising concerns, or admitting mistakes without fear of negative consequences (Edmondson, 1999). A substantial body of research has linked team psychological safety to improved communication, enhanced learning, better performance, and reduced stress within healthcare teams (Frazier et al., 2017; O’Donovan et al., 2020). However, team psychological safety represents only one dimension of workers’ experience; it does not fully capture how individuals internally detect and process cues of safety or threat.

Psychological safety has most commonly been conceptualised through interpersonal and organisational lenses, particularly at the team level, where it is understood as a shared belief that the team is safe for interpersonal risk-taking. Cognitive–affective approaches have also emphasised individuals’ appraisals of threat and safety within work environments. In this study, Polyvagal Theory is used to complement these perspectives by foregrounding the embodied, neuroceptive processes through which individuals experience safety. This framing is particularly relevant for health and social care workers, whose roles often involve sustained stress and emotional labour, and allows individual psychological safety to be examined alongside established team-level constructs within a single model.

Polyvagal Theory (PVT; Porges, 2011, 2024) offers a neurophysiological framework for understanding psychological safety at the individual level. According to this theory, the autonomic nervous system continuously engages in *neuroception*, the unconscious detection of cues of danger or safety which shapes emotional regulation, social engagement, and stress responses. Drawing on this framework, the Neuroception of Psychological Safety Scale (NPSS) was developed to measure embodied experiences of safety through three dimensions: social engagement, compassion, and bodily sensations (Morton et al., 2024). These dimensions capture internal felt states that support co-regulation, openness, and adaptive responding mechanisms that may be particularly relevant for HSCWs frequently exposed to trauma, unpredictability, and high emotional labour (Cogan et al., 2024; Melander et al., 2024).

Trauma-informed approaches similarly emphasise the importance of felt safety, noting that structural protections alone are insufficient if individuals do not physiologically experience safety (Domínguez-Salas et al., 2021; Roche et al., 2025). In frontline settings, both individual neuroceptive processes and team relational dynamics may play essential roles in shaping resilience. Individual safety may support emotional regulation and personal coping, while team-level safety may provide collective buffering during demanding or distressing events (Amoadu et al., 2025). Despite growing interest in both levels of psychological safety, few studies have examined them simultaneously or explored how they jointly contribute to well-being, burnout, and acute stress in health and social care workforces. This observation is specific to studies that examine individual-level psychological safety alongside team-level psychological safety within the same analytic model. While team psychological safety has been widely studied, particularly within organisational and healthcare settings, far fewer studies have integrated individual, embodied experiences of safety with team-level constructs when examining well-being and stress-related outcomes.

To address this gap, the present study explores the role of psychological safety at both the individual and team levels among a large sample of HSCWs. Specifically, it examines how neuroceptive experiences of safety and team-based relational safety are associated with well-being, burnout, and acute stress. Rather than positioning individual and team psychological safety in competition, the aim of this study is to develop a more comprehensive understanding of how multiple dimensions of psychological safety operate within frontline care environments. By examining both constructs simultaneously, this research seeks to clarify the mechanisms through which psychological safety may protect against distress, support resilience, and contribute to workforce sustainability. In the present study, both psychological safety constructs are examined at the individual level, with team psychological safety reflecting individuals’ perceptions of safety within their team context rather than an aggregated team-level climate in the multilevel sense defined by Edmondson (1999). To enhance conceptual clarity, this study was guided by the following research questions:

1. To what extent do individual psychological safety (as measured by neuroceptive experiences of safety) and team psychological safety independently predict mental well-being, burnout, and acute stress among health and social care workers?
2. Does team psychological safety explain additional variance in these outcomes beyond individual psychological safety?
3. How do the relative contributions of individual and team psychological safety differ across well-being, burnout, and acute stress outcomes?

Drawing on Polyvagal Theory, individual psychological safety is conceptualised as reflecting neuroceptive processes that support autonomic regulation, social engagement, and stress recovery. From this perspective, a greater embodied sense of safety is expected to be associated with lower acute stress and burnout, and with higher psychological well-being. In parallel, team-based psychological safety can be understood through organisational and job demand–resource frameworks as a contextual resource that reduces interpersonal threat, facilitates support-seeking, and buffers against chronic occupational strain. Together, these perspectives provide a theoretical basis for examining how general neuroceptive safety and team-specific safety are differentially associated with stress-related and well-being outcomes among health and social care workers.

Based on this theoretical framework, the following hypotheses were tested. First, higher levels of individual psychological safety (neuroceptive safety) and team psychological safety were hypothesised to be associated with better mental well-being and lower levels of burnout and acute stress. Second, team psychological safety was hypothesised to explain additional variance in well-being, burnout, and acute stress beyond individual psychological safety. Finally, it was hypothesised that the relative contributions of individual and team psychological safety would differ across outcomes, with team psychological safety showing a stronger association with burnout and acute stress.

## 2. Method

This study employed a predictive correlational design to examine associations between psychological safety and mental health outcomes among health and social care workers (HSCWs). This design is appropriate for assessing the strength and direction of relationships between theoretically relevant constructs and for identifying predictors of acute stress, burnout, and well-being in occupational settings. Predictive correlational approaches have been widely used in occupational and trauma-informed research to examine antecedents of stress-related outcomes in frontline populations (Domínguez-Salas et al., 2021).

In the present study, individual psychological safety (as measured by neuroceptive experiences of safety) and perceived team psychological safety were treated as the primary independent variables. Acute stress, mental well-being, and burnout were specified as dependent variables, each assessed using validated psychometric measures. All variables were analysed at the individual level, reflecting participants’ self-reported perceptions and experiences.

### 2.1. Materials

The Neuroception of Psychological Safety Scale (NPSS; Morton et al., 2024) is a 29-item self-report instrument designed to assess individual perceptions of psychological safety, grounded in Polyvagal Theory. The NPSS conceptualises psychological safety as an embodied, neuroceptive experience and measures it across three interrelated dimensions: social engagement, compassion, and bodily sensations.

The *social engagement* dimension captures feelings of acceptance, trust, and openness within social contexts, reflecting an individual’s capacity to engage safely with others. The *compassion* dimension reflects empathy, care, and a willingness to support others, while the *bodily sensations* dimension assesses internal physiological states—such as a steady heart rate, relaxed muscles, and a general sense of physical calm—indicative of autonomic regulation. Collectively, the NPSS assesses individuals’ embodied experiences of safety and threat within occupational and social environments, capturing felt safety, physiological regulation, and capacity for social engagement. Example items include “*I feel safe to be myself at work*” and “*My body feels calm and settled in my work environment*.”

Participants rate each item on a 5-point Likert scale ranging from *strongly disagree* to *strongly agree*, with higher scores indicating greater perceived psychological safety. Participants were instructed to respond with reference to their general experiences of safety across social and occupational contexts, rather than focusing on a single team or situation. This approach aligns with the intended use of the NPSS as a context-transcending measure of neuroceptive safety, capturing individuals’ embodied sense of safety across interpersonal and work-related settings. The NPSS has demonstrated strong internal consistency across multiple studies, supporting its reliability as a measure of individual psychological safety (Cogan et al., 2024, 2025; Poli & Miccoli, 2024).

The TPSS (Edmondson, 1999), is a 7-item scale that assesses the collective belief among team members that the team environment is safe for interpersonal risk-taking. The TPSS includes statements such as “If you make a mistake on this team, it is often held against you” and “It is safe to take a risk on this team.” Participants respond on a 7-point Likert scale ranging from “very inaccurate” to “very accurate.” Higher scores reflect a stronger perception of team-based psychological safety. The TPSS has been extensively validated in various organisational contexts and is associated with positive outcomes like team collaboration and innovation (Edmondson & Lei, 2014).

The Short Warwick–Edinburgh Mental Well-being Scale (SWEMWBS; Ng Fat et al., 2017) is a brief, 7-item scale that measures overall mental well-being by assessing positive mental states like optimism, relaxation, and confidence. The SWEMWBS includes statements such as “I’ve been feeling useful” and “I’ve been feeling close to other people.” Responses are given on a 5-point scale from “none of the time” to “all of the time,” with higher scores reflecting better well-being and capturing an overview of emotional and psychological health. The scale is known for its high reliability (Ng Fat et al., 2017) and has been validated across diverse populations, proving to be a sensitive tool for measuring changes in mental health.

The Burnout Measure—Short Version (BMS; Malach-Pines, 2005) is a 10-item scale that assesses burnout across physical, emotional, and mental dimensions. Items cover symptoms like fatigue, worthlessness, and emotional exhaustion, with statements such as “I’ve had it” and “I feel emotionally drained.” Responses are rated on a 7-point scale from “never” to “always,” with higher scores indicating higher burnout levels. The BMS is widely used in occupational health research for identifying levels of burnout (Riley et al., 2018), making it a validated and effective measure for assessing burnout across various professional and personal contexts.

The Abbreviated Post-Traumatic Stress Disorder Checklist—Civilian (APCL-C; Lang & Stein, 2005) is a 6-item measure designed to assess acute stress symptoms in civilian populations. It evaluates typical trauma responses such as re-experiencing, hyperarousal, and avoidance. Items include “Repeated, disturbing memories, thoughts, or images of a stressful experience from the past,” with responses on a 5-point Likert scale from “not at all” to “extremely.” Higher scores indicate greater levels of acute stress, suggesting potential need for further mental health support. The APCL-C has demonstrated good internal consistency and is widely used in assessing acute stress symptoms in non-combat populations (Freedy et al., 2010).

In the present sample, all measures demonstrated good to excellent internal consistency. Cronbach’s alpha was α = 0.94 for the Neuroception of Psychological Safety Scale (NPSS), α = 0.88 for the Team Psychological Safety Scale (TPSS), α = 0.89 for the Short Warwick–Edinburgh Mental Well-being Scale, α = 0.92 for the Burnout Measure—Short Version, and α = 0.86 for the Acute Stress measure.

### 2.2. Procedure and Recruitment

Participants were recruited through NHS organisational networks, professional associations, and social media advertisements targeting health and social care workers. The study was promoted via social media platforms, relevant forums, and websites focused on mental health and well-being, providing a link to the survey and encouraging eligible individuals to participate. Additionally, NHS departments, health and social care employers, and professional bodies shared the study within their staff networks to reach workers who met the eligibility criteria.

The survey, administered via the online platform Qualtrics, began with a participant information sheet followed by an online consent form. Participants were then asked to provide socio-demographic information before completing a series of validated psychological measures. These included the NPSS, TPSS, SWEMWBS, BMS and the APCL-C. The survey was designed to be anonymous, with each participant assigned a unique identifier to ensure privacy. The study’s inclusion criteria focused on adults aged 18 and older, working in a health and social care workplace setting for a minimum of 6 months, ensuring that data gathered would be relevant to the study’s objectives. Exclusion criteria, such as dementia, severe drug or alcohol dependency, active psychosis, or neuropsychiatric conditions requiring hospitalisation, were applied to help ensure participants had the capacity to provide informed consent and to maintain the reliability of self-reported data. This careful selection process aimed to capture a representative sample of the target population while excluding individuals whose specific conditions could limit accurate self-report.

The survey was structured to encourage honest and open responses, and it included a debrief section at the end, providing participants with information on available mental health support services such as helplines, online counselling resources, and local community organisations. This was particularly important given the nature of the survey, which dealt with sensitive issues like acute stress and mental wellbeing. Participants were assured of the confidentiality of their responses, which were anonymous with no personally identifiable information collected.

### 2.3. Ethics

Ethical approval for the study was obtained from the University Ethics Committee (Ref: 33/02/12/2020/A). Informed consent was obtained from all participants, who were given a clear explanation of the study’s purpose, the voluntary nature of their participation. All data were securely stored on the University server, and no personally identifiable information was collected or linked to participants’ responses. The findings were reported in aggregate form to further protect participant identities. The study adhered to the British Psychological Society’s ethical guidelines for research involving potentially vulnerable populations, ensuring participants’ well-being was prioritised throughout.

### 2.4. Analysis

Correlational and multiple regression analyses were conducted to examine the predictive role of psychological safety measured via individual psychological safety (NPSS) and team psychological safety (TPSS) in relation to well-being, burnout, and acute stress. As data were collected across heterogeneous settings without team identifiers, team psychological safety was analysed as an individual-level perception of the team environment, and no aggregation or intraclass correlation analyses were conducted. Prior to analysis, the dataset was screened for missing values. Missing data were minimal and handled using pairwise deletion for correlations and listwise deletion within each regression model, consistent with recommended practices for large samples. Assumption checks were conducted before running the regressions. Tests of normality indicated significant deviations at *p* < 0.05, which is common in large datasets; however, visual inspection of histograms and Q–Q plots, along with skewness and kurtosis values, indicated that the variables were sufficiently normally distributed for parametric analysis. Given the large sample size (*n* = 821), minor deviations from normality were not considered problematic. Multicollinearity was assessed using Variance Inflation Factors (VIFs), all of which were below 2, indicating no multicollinearity concerns between NPSS and TPSS. Examination of standardised residuals and residual scatterplots confirmed that assumptions of homoscedasticity and linearity were met. Residual distributions also supported the assumption of normality. These checks indicated that the data were appropriate for regression analysis. Scale scores were computed as the sum of item responses. This approach was selected to remain consistent with the original scale development and validation procedures and to preserve the full variability of the underlying constructs. Summed scores are mathematically equivalent to averaged scores when item counts are constant; however, summed scoring maintains the original metric of the measures and facilitates interpretation of regression coefficients across outcomes.

## 3. Results

### 3.1. Participants

The sample consisted of 821 HSCWs with a mean age of 38.65 years (SD = 11.40). The majority identified as female (70.0%), and most identified as White (74.2%), with additional representation from Black African (14.4%), Asian (10.2%), and mixed ethnicity (1.1%) groups. Most participants lived in urban areas (70.8%) and were employed full-time (65.7%). Regarding occupational background, the largest proportion of respondents were nurses, followed by doctors/advanced clinical staff, allied health professionals, health and social care support staff, and mental health professionals. Collectively, these frontline groups represented over half of the sample, indicating strong representation from direct care roles. Workplace seniority varied, with 25.0% identifying as entry level, 46.5% as intermediate, 26.5% as senior, and 2.0% as executive, reflecting a broad distribution of responsibilities across the sector. Exposure to occupational trauma was highly prevalent: 44.5% reported direct exposure, 15.3% indirect exposure, and 25.2% both, meaning that 80.2% of the sample had encountered some form of traumatic experience at work. Notably, 44.5% reported that such exposure had adversely affected their psychological functioning. Detailed participant characteristics are shown in Table 1.

The results presented in Table 2 offer a comprehensive overview of psychological safety, stress, well-being, and burnout among health and social care workers (HSCWs). Individual psychological safety, measured by the NPSS, showed a moderately high average score, suggesting that workers generally feel valued, understood, and emotionally supported at the individual level. In contrast, team psychological safety (TPSS) demonstrated a lower average score, indicating that while some team-based support and cohesion are present, the team environment is notably less safe and consistent than individual relational experiences. Acute stress scores were moderate, reflecting the ongoing exposure to emotionally demanding and safety-critical situations in frontline care. Burnout levels were also moderate to high, highlighting significant emotional exhaustion and strain across the workforce. Mental well-being, as measured by the SWEMWBS, fell in the moderate range, suggesting that although a portion of staff maintain positive psychological functioning, a considerable proportion experience reduced well-being likely linked to chronic workload pressures and insufficient recovery opportunities. These findings reveal a workforce experiencing elevated levels of stress and burnout, accompanied by only moderate well-being, despite relatively strong individual feelings of psychological safety. The lower levels of team psychological safety are especially noteworthy, as they may signal inconsistent support, communication, or emotional buffering within teams.

The correlation analysis presented in Table 3 revealed robust associations among psychological safety, well-being, burnout, and acute stress. Individual psychological safety (NPSS) showed a strong positive association with mental well-being (r = 0.68, *p* < 0.01), indicating that workers who felt personally safe, valued, and understood reported significantly better psychological health. NPSS was also moderately negatively correlated with burnout (r = −0.46, *p* < 0.01) and acute stress (r = −0.38, *p* < 0.01), suggesting that greater individual psychological safety was linked to lower emotional exhaustion and fewer acute stress symptoms

Team psychological safety (TPSS) demonstrated a strong correlation with NPSS (r = 0.83, *p* < 0.01), reflecting substantial overlap between personal and team-based safety experiences, although the two constructs remain distinct. TPSS was also strongly positively associated with mental well-being (r = 0.68, *p* < 0.01) and moderately negatively associated with both burnout (r = −0.59, *p* < 0.01) and acute stress (r = −0.54, *p* < 0.01). Notably, TPSS showed stronger associations with burnout and acute stress than NPSS, underscoring the influential role of team dynamics in shaping frontline workers’ psychological outcomes. Mental well-being itself demonstrated strong negative correlations with both burnout (r = −0.73, *p* < 0.01) and acute stress (r = −0.48, *p* < 0.01), confirming that lower distress is closely tied to higher well-being. Burnout and acute stress were strongly positively correlated (r = 0.70, *p* < 0.01), reflecting the interconnected nature of short-term stress responses and longer-term emotional exhaustion. These findings highlight that while both individual and team psychological safety function as important protective factors, team psychological safety shows the strongest and most consistent associations with reduced burnout and acute stress, emphasising the central role of supportive team environments in safeguarding psychological health among health and social care workers.

### 3.2. Regression Analysis

Hierarchical multiple regression analyses were conducted to examine the relative contributions of individual psychological safety (NPSS) and team psychological safety (TPSS) in predicting mental well-being, burnout, and acute stress. NPSS was entered at Step 1, followed by TPSS at Step 2 to assess its incremental predictive value. For mental well-being, NPSS entered at Step 1 accounted for a substantial proportion of variance (46%). The addition of TPSS at Step 2 produced a significant improvement in model fit, ΔR^2^ = 0.04, indicating that team psychological safety explained an additional 4% of the variance in well-being beyond individual psychological safety. In the final model, both NPSS (β = 0.38, *p* < 0.001) and TPSS (β = 0.36, *p* < 0.001) were significant predictors and showed comparable effect sizes. For burnout, NPSS explained 21% of the variance at Step 1. When TPSS was included, the model showed a significant and notable increase in explanatory power, with ΔR^2^ = 0.14. In the final model, TPSS emerged as a strong and statistically significant predictor of burnout (β = 0.67, *p* < 0.001), whereas NPSS was weaker and no longer significant once team safety was accounted for (β = −0.09, ns). For acute stress, NPSS explained 27% of the variance at Step 1. The inclusion of TPSS at Step 2 resulted in a further significant increase in explained variance, ΔR^2^ = 0.16, demonstrating that team psychological safety contributed an additional 16% of explanatory power. In the final model, both predictors were significant, with TPSS showing a markedly stronger association with acute stress (β = 0.72, *p* < 0.001) than NPSS (β = −0.22, *p* < 0.001). Across all three outcomes, team psychological safety accounted for substantially more variance than individual psychological safety. Although NPSS contributed meaningfully in Step 1 models, the inclusion of TPSS consistently improved model performance, indicating that team-level relational safety plays a central role in predicting well-being, burnout, and acute stress among health and social care workers.

For clarity, higher scores on the TPSS reflect greater perceived psychological safety. Accordingly, the positive standardized coefficients reported in Table 4 indicate that higher team psychological safety is associated with lower levels of burnout and acute stress when considered alongside individual psychological safety, consistent with the zero-order correlations and the theoretical framing of psychological safety as a protective factor.

## 4. Discussion

This study examined the predictive role of individual and team psychological safety in relation to well-being, acute stress, and burnout among health and social care workers. The findings demonstrated that although both individual psychological safety and perceived team psychological safety were significantly associated with mental health outcomes, team-based psychological safety emerged as the stronger and more consistent predictor, particularly for burnout and acute stress. These results challenge assumptions that individual psychological safety alone is the primary protective factor and instead highlight the central importance of relational and interpersonal dynamics within team contexts. It is important to note that, consistent with the study design, the present findings reflect individual perceptions of psychological safety rather than aggregated team-level climate, and therefore do not make claims about team psychological safety as a shared group property in the multilevel sense. From a Polyvagal Theory perspective, the NPSS provides a unique lens through which to understand individual experiences of safety. The NPSS captures neuroceptive cues, the body’s automatic detection of threat or safety, through dimensions of social engagement, compassion, and embodied regulation (Porges, 2024; Morton et al., 2024). These neurophysiological states are critical in high-demand environments where HSCWs face emotional labour, trauma exposure, and cumulative stressors that can dysregulate the autonomic nervous system (Cogan et al., 2024; Melander et al., 2024; Peddie et al., 2025). NPSS therefore remains an important indicator of individual vulnerability and resilience, offering insight into how workers internally process safety signals that may buffer against stress.

However, the current findings clearly show that team-based psychological safety exerts a more powerful influence on psychological outcomes. TPSS demonstrated strong correlations with well-being and robust negative associations with burnout and acute stress. In the regression models, TPSS accounted for substantially more variance than NPSS in predicting both burnout and acute stress, with large effect sizes. This aligns with polyvagal and trauma-informed frameworks, which emphasise that safety is fundamentally co-regulated. The autonomic nervous system is highly sensitive to social cues, and team environments provide the most immediate signals regarding predictability, support, and relational protection. (Porges, 2025). In frontline settings, distress rarely emerges in isolation; it is shaped by collective workload pressures, shared exposure to traumatic incidents, communication patterns, and the emotional availability of colleagues (Griffith et al., 2023). Supportive, psychologically safe teams can diffuse acute stress, distribute emotional load, and prevent the build-up of chronic strain. Conversely, team environments marked by conflict, blame, or inconsistency may amplify threat signals, accelerating burnout and dysregulation (Rott et al., 2025). These findings therefore highlight the need to conceptualise psychological safety in HSCWs not only as an individual phenomenon but as a relational and systemic resource embedded within team functioning.

The strong correlation observed between individual neuroceptive psychological safety (NPSS) and team psychological safety (TPSS) warrants brief consideration. Although highly related, these constructs are conceptually distinct. NPSS reflects an individual’s embodied, neuroceptive sense of safety grounded in Polyvagal Theory, whereas TPSS captures shared perceptions of interpersonal risk-taking and safety within teams. Some degree of overlap is theoretically expected, particularly in frontline contexts where team environments may shape individuals’ physiological and psychological experiences of safety. Importantly, the differential patterns of association observed across outcomes—most notably the stronger predictive role of TPSS for burnout and acute stress—support the discriminant validity of the two constructs and suggest that they capture related but non-redundant dimensions of psychological safety operating at different levels.

The NPSS still offers important added value. Although team experiences dominated prediction for burnout and acute stress, NPSS remained significantly associated with well-being and contributed additional nuance to the understanding of individual differences in sensitivity to threat or safety. This neuroceptive dimension may help explain why within the same team some individuals exhibit higher vulnerability or greater resilience (Peddie et al., 2025). Team-level surveys alone cannot capture this embodied variability, making NPSS a valuable complement to existing organisational metrics.

One limitation of the present study is that key occupational and trauma-related factors, such as role type, seniority, workload intensity, or cumulative exposure to traumatic events, were not included as covariates in the analyses. These factors are known to influence psychological outcomes in health and social care workers and may interact with experiences of psychological safety. The decision not to include these variables was intentional, reflecting the study’s primary aim to examine the independent and incremental contributions of individual (neuroceptive) and perceived team psychological safety. Future research would benefit from incorporating occupational characteristics and trauma exposure alongside psychological safety constructs to better understand how these factors jointly shape well-being, burnout, and acute stress in frontline workforces.

The findings should also be interpreted in light of the study’s cross-sectional design and exclusive reliance on self-report measures. As data were collected at a single time point using the same method, the potential for common method variance cannot be fully ruled out. Although validated instruments were used and the pattern of results showed differential associations across outcomes, causal inferences cannot be drawn. Longitudinal and multi-method research designs, including objective indicators or observer-rated measures, would help to clarify temporal relationships and reduce shared method bias in future studies. The implications of these findings are particularly salient in the context of ongoing workforce crises in health and social care. International evidence indicates rising absenteeism, turnover, and dissatisfaction (Fronteira et al., 2024; Jun et al., 2021; Mahat et al., 2025), with burnout a major driver of attrition. The present results suggest that interventions designed to strengthen psychological safety should prioritise the team relationship climate, particularly enhancing trust, communication, and co-regulation. However, this does not diminish the value of individual-focused supports. Programmes that foster social engagement, compassion, and awareness of bodily safety cues, core NPSS dimensions, may offer additional protection against chronic stress, particularly for workers with heightened neuroceptive sensitivity. An integrated approach that strengthens both individual neuroceptive safety and team-level interpersonal safety is therefore likely to offer the greatest benefit. This dual focus aligns with contemporary trauma-informed frameworks, which emphasise that staff require environments where they both *are safe* and *feel safe* on a physiological and relational level (Domínguez-Salas et al., 2021; Roche et al., 2025).

## 5. Conclusions

This study demonstrated that psychological safety plays a central role in shaping the well-being, stress, and burnout of health and social care workers. Although individual psychological safety was significantly associated with all outcomes, team psychological safety emerged as the strongest predictor of burnout and acute stress, underscoring the fundamentally relational nature of psychological safety in high-pressure care environments. These results highlight that workforce well-being is not solely dependent on individual resilience but is profoundly shaped by the quality of team interactions, shared support, and interpersonal trust. Organisational interventions should therefore prioritise strengthening team psychological safety as a core strategy for preventing burnout and reducing acute stress. At the same time, NPSS offers a sensitive and theoretically grounded tool for identifying individual neuroceptive vulnerability or resilience, complementing team-based approaches. By embedding both NPSS and TPSS within routine workforce assessment, health and social care systems may be better equipped to detect risk early, tailor support, and promote sustainable, compassionate, and resilient workplace cultures. These findings align with prior work highlighting the importance of psychological safety for workforce well-being and functioning in high-demand healthcare environments (Edmondson & Lei, 2014; Greenberg et al., 2020). By demonstrating the distinct contributions of individual neuroceptive safety and team psychological safety, this study extends existing multilevel approaches to understanding stress, burnout, and well-being among health and social care workers.

## Figures and Tables

**Table 1:** Participant Characteristics for Health & Social Care Workers.

Variable	*n* (%)
**Age**	*M* = 38.65, *SD* = 11.40 (valid *n* = 809)
**Gender**	
Female	574 (70.0%)
Male	240 (29.3%)
Non-binary	6 (0.7%)
**Ethnicity**	
White (British/Other)	594 (74.2%)
Black African	115 (14.4%)
Asian	82 (10.2%)
Mixed/Multiple	9 (1.1%)
**Community type**	
Urban	569 (70.8%)
Rural	206 (25.6%)
Other	29 (3.6%)
**Employment status**	
Full-time	539 (65.7%)
Part-time	235 (28.6%)
Student	22 (2.7%)
Voluntary work	8 (1.0%)
Carer	7 (0.9%)
**Seniority**	
Entry level	198 (25.0%)
Intermediate	368 (46.5%)
Senior	210 (26.5%)
Executive	16 (2.0%)
**Exposure to trauma at work**	
Direct	347 (44.5%)
Indirect	119 (15.3%)
Both	196 (25.2%)
None	117 (15.0%)
**Adverse impact of trauma on self**	
Yes	351 (44.5%)
No	328 (41.6%)
Don’t know	109 (13.8%)

*Note***.** Valid percentages are shown for each variable. Missing data: Age (*n* = 12), Gender (*n* = 1 excluded for non-valid categories), Ethnicity (*n* = 21 non-classifiable categories excluded), Community (*n* = 17), Employment (*n* = 0), Seniority (*n* = 29), Trauma Exposure (*n* = 42), Trauma Impact (*n* = 33).

**Table 2:** Descriptive Statistics of Psychometric Measures.

Measure	M	SD
Mental well-being (SWEMWBS)	24.79	10.57
Burnout (BMS)	95.23	45.83
Acute stress (APCL-C)	12.84	6.97
Individual psychological safety (NPSS)	98.52	35.65
Team psychological safety (TPSS)	25.78	9.39

*Note*. Scale scores represent the sum of all items within each measure. Higher scores indicate better wellbeing (SWEMWBS), higher burnout (BMS), greater acute stress (PCL-6), and greater perceived psychological safety (NPSS/TPSS). All descriptive statistics are based on valid responses (*n* = 821).

**Table 3:** Correlation Matrix for Key Study Variables.

Measure	1	2	3	4	5
1. Mental Wellbeing (SWEMWBS)	—				
2. Burnout (BMS)	−0.73 **	—			
3. Acute Stress (PCL-6)	−0.48 **	0.70 **	—		
4. NPSS (Individual Safety)	0.68 **	−0.46 **	−0.38 **	—	
5. TPSS (Team Safety)	0.68 **	−0.59 **	−0.54 **	0.83 **	—

*Note*. NPSS = Neuroception of Psychological Safety Scale (29 items). TPSS = Team Psychological Safety Scale (7 items). SWEMWBS = Short Warwick–Edinburgh Mental Wellbeing Scale. PCL-6 = Acute Stress. BMS = Burnout Measure. All correlations are significant at ** *p* < 0.01.

**Table 4:** Hierarchical Regression Analyses Predicting Well-being, Burnout, and Acute Stress.

Predictor	Well-Being (β)	Burnout (β)	Acute Stress (β)
NPSS	0.38 *	−0.09	−0.22 ***
TPSS	0.36 *	0.67 *	0.72 *
R^2^ (final model)	0.50	0.35	0.30
ΔR^2^ (TPSS after NPSS)	0.04 ***	0.14 ***	0.16 ***

*Note*. Entries are standardised regression coefficients (β) from the final models. R^2^ = variance explained by the full model (NPSS + TPSS). ΔR^2^ = additional variance explained when TPSS was added after NPSS (Step 2 vs. Step 1). NPSS = Neuroception of Psychological Safety Scale (29 items); TPSS = Team Psychological Safety Scale (7 items); SWEMWBS = Short Warwick–Edinburgh Mental Wellbeing Scale; PCL-6 = Acute Stress; BMS = Burnout Measure. * *p* < 0.05, *** *p* < 0.001.

## Data Availability

The data supporting the findings of this study are stored securely within the University of Strathclyde research repository. Due to privacy and ethical restrictions, the dataset cannot be made publicly available. Data may be accessed upon reasonable request by contacting the corresponding author, provided that such access complies with ethical and confidentiality requirements.

## References

[B1-behavsci-16-00418] Amoadu M., Agyare D. F., Doe P. F., Abraham S. A. (2025). Examining the impact of psychosocial safety climate on working conditions, well-being and safety of healthcare providers: A scoping review. BMC Health Services Research.

[B2-behavsci-16-00418] Cogan N., Archbold H., Deakin K., Griffith B., Sáez Berruga I., Smith S., Tanner G., Flowers P. (2022a). What have we learned about what works in sustaining mental health care and support services during a pandemic? Transferable insights from the COVID-19 response within the NHS Scottish context. International Journal of Mental Health.

[B3-behavsci-16-00418] Cogan N., Campbell J., Morton L., Young D., Porges S. (2024). Validation of the neuroception of psychological safety scale (NPSS) among health and social care workers in the UK. International Journal of Environmental Research and Public Health.

[B4-behavsci-16-00418] Cogan N., Kennedy C., Beck Z., McInnes L., MacIntyre G., Morton L., MacIntyre G., Morton L., Tanner G., Kolacz J. (2022b). ENACT study: What has helped health and social care workers maintain their mental well-being during the COVID-19 pandemic?. Health & Social Care in the Community.

[B5-behavsci-16-00418] Cogan N., Morton L., Campbell J., Irvine Fitzpatrick L., Lamb D., De Kock J., Ali A., Young D., Porges S. (2025). Neuroception of psychological safety scale (NPSS): Validation with a UK based adult community sample. European Journal of Psychotraumatology.

[B6-behavsci-16-00418] Domínguez-Salas S., Gómez-Salgado J., Guillén-Gestoso C., Romero-Martín M., Ortega-Moreno M., Ruiz-Frutos C. (2021). Health care workers’ protection and psychological safety during the COVID-19 pandemic in Spain. Journal of Nursing Management.

[B7-behavsci-16-00418] Edmondson A. (1999). Psychological safety and learning behavior in work teams. Administrative Science Quarterly.

[B8-behavsci-16-00418] Edmondson A., Lei Z. (2014). Psychological safety: The history, renaissance, and future of an interpersonal construct. Annual Review of Organizational Psychology and Organizational Behavior.

[B9-behavsci-16-00418] Frazier M. L., Fainshmidt S., Klinger R. L., Pezeshkan A., Vracheva V. (2017). Psychological safety: A meta-analytic review and extension. Personnel Psychology.

[B10-behavsci-16-00418] Freedy J. R., Steenkamp M. M., Magruder K. M., Yeager D. E., Zoller J. S., Hueston W. J., Carek P. J. (2010). Post-traumatic stress disorder screening test performance in civilian primary care. Family Practice.

[B11-behavsci-16-00418] Fronteira I., Mathews V., dos Santos R. L. B., Matsumoto K., Amde W., Pereira A., de Oliveira A. P. C., Craveiro I., Chança R., Boniol M., Ferrinho P., Poz M. R. D. (2024). Impacts for health and care workers of COVID-19 and other public health emergencies of international concern: Living systematic review, meta-analysis and policy recommendations. Human Resources for Health.

[B12-behavsci-16-00418] Greenberg N., Docherty M., Gnanapragasam S., Wessely S. (2020). Managing mental health challenges faced by healthcare workers during covid-19 pandemic. BMJ.

[B13-behavsci-16-00418] Griffith B., Archbold H., Saez Berruga I., Smith S., Deakin K., Cogan N., Tanner G., Flowers P. (2023). Frontline experiences of delivering remote mental health supports during the COVID-19 pandemic in Scotland: Innovations, insights and lessons learned from mental health workers. Psychology, Health & Medicine.

[B14-behavsci-16-00418] Jun J., Ojemeni M. M., Kalamani R., Tong J., Crecelius M. L. (2021). Relationship between nurse burnout, patient and organizational outcomes: Systematic review. International Journal of Nursing Studies.

[B15-behavsci-16-00418] Lang A. J., Stein M. B. (2005). An abbreviated PTSD checklist for use as a screening instrument in primary care. Behaviour Research and Therapy.

[B16-behavsci-16-00418] Mahat S., Lehmusto H., Rafferty A. M., Vehviläinen-Julkunen K., Mikkonen S., Härkänen M. (2025). Impact of second victim distress on healthcare professionals’ intent to leave, absenteeism and resilience: A mediation model of organizational support. Journal of Advanced Nursing.

[B17-behavsci-16-00418] Malach-Pines A. (2005). The Burnout Measure, short version. International Journal of Stress Management.

[B18-behavsci-16-00418] Melander S., Dahl O., Falk A. C., Lindström V., Andersson E., Gustavsson P., Rudman A. (2024). Critical incidents and post-traumatic stress symptoms among experienced registered nurses during the COVID-19 pandemic: A cross-sectional study. International Journal of Nursing Studies Advances.

[B19-behavsci-16-00418] Morton L., Cogan N., Kolacz J., Calderwood C., Nikolic M., Bacon T., Pathe E., Williams D., Porges S. W. (2024). A new measure of feeling safe: Developing psychometric properties of the neuroception of psychological safety scale (NPSS). Psychological Trauma-US.

[B20-behavsci-16-00418] Ng Fat L., Scholes S., Boniface S., Mindell J., Stewart-Brown S. (2017). Evaluating and establishing national norms for mental wellbeing using the Short Warwick–Edinburgh Mental Well-Being Scale (SWEMWBS): Findings from the health survey for England. Quality of Life Research.

[B21-behavsci-16-00418] O’Donovan R., Van Dun D., McAuliffe E. (2020). Measuring psychological safety in healthcare teams: Developing an observational measure to complement survey methods. BMC Medical Research Methodology.

[B22-behavsci-16-00418] Peddie N., Hoegh J., Rice G., Shetty S., Ure A., Cogan N. (2025). Health and social care professionals’ experience of psychological safety within their occupational setting: A thematic synthesis review. Nursing Reports.

[B23-behavsci-16-00418] Poli A., Miccoli M. (2024). Validation of the Italian version of the Neuroception of Psychological Safety Scale (NPSS). Heliyon.

[B24-behavsci-16-00418] Porges S. W. (2011). The polyvagal theory: Neurophysiological foundations of emotions, attachment, communication, and self-regulation.

[B25-behavsci-16-00418] Porges S. W. (2024). Polyvagal Theory: The neuroscience of safety in trauma-informed practice. The handbook of trauma-transformative practice: Emerging therapeutic frameworks for supporting individuals, families or communities impacted by abuse and violence.

[B26-behavsci-16-00418] Porges S. W. (2025). Polyvagal theory: Current status, clinical applications, and future directions. Clinical Neuropsychiatry.

[B27-behavsci-16-00418] Riley M. R., Mohr D. C., Waddimba A. C. (2018). The reliability and validity of three-item screening measures for burnout: Evidence from group-employed health care practitioners in upstate New York. Stress and Health.

[B28-behavsci-16-00418] Roche H., Morton L., Cogan N. (2025). Barriers and facilitators to psychological safety during medical procedures among individuals diagnosed with chronic illness in childhood. Healthcare.

[B29-behavsci-16-00418] Rott C., Segers M., Van den Bossche P. (2025). Expected to be calm in any storm: An exploration of the stress experiences of crisis team leaders. International Journal of Disaster Risk Reduction.

